# Total knee arthroplasty in femoral bowing: does patient specific instrumentation have something to add? A randomized controlled trial

**DOI:** 10.1186/s12891-021-04198-5

**Published:** 2021-04-02

**Authors:** Sammy Abdullah ALShammari, Keun Young Choi, In Jun Koh, Man Soo Kim, Yong In

**Affiliations:** 1 Department of Orthopaedic Surgery, Seoul St. Mary’s Hospital, College of Medicine, The Catholic University of Korea, 222 Banpo-Daero, Seocho-Gu, Seoul, 06591 Republic of Korea; 2 Department of Orthopaedic Surgery, Eunpyeong St. Mary’s Hospital, College of Medicine, The Catholic University of Korea, Seoul, Republic of Korea

**Keywords:** Total knee arthroplasty, Femoral bowing, Alignment, Component position, Patient specific instrument

## Abstract

**Background:**

Patient-specific instrumentation (PSI) proponents have suggested the benefits of improved component alignment and reduced outliers. In this randomized controlled trial, we attempted to assess the advantage of using PSI over conventional intermedullary (IM) guides for primary total knee arthroplasty (TKA) with bilateral severe femoral bowing (> 5°). A parallel trial design was used with 1:1 allocation. We hypothesize that PSI would support more accurate alignment of components and the lower-limb axis during TKA with severe femoral bowing in comparison with conventional IM guides.

**Methods:**

Among 336 patients undergoing bilateral TKAs due to knee osteoarthritis, 29 patients with bilateral lateral femoral bowing of more than 5° were included in this study. Every patient was assigned randomly to PSI on one side and to conventional instrumentation lateralization of the entry point of the femoral IM guide was applied on the other with a goal of neutral mechanical alignment. The assessment of coronal alignment was completed by measuring the hip–knee–ankle (HKA) angle on preoperative and postoperative long film standing radiographs. Coronal and sagittal orientations of femoral and tibial components were assessed on weight-bearing radiographs. The rotational alignment of the femoral component was evaluated using computed tomography.

**Results:**

The postoperative mean ± standard deviation (SD) HKA angle was varus 4.0° (± 2.7°) for conventional technique and varus 4.1° (± 3.1°) for PSI, with no differences between the two groups (*p* = 0.459). The component orientation showed no significant differences except with respect to the sagittal alignment of the femoral component (*p* = 0.001), with a PSI mean ± SD flexion of 5.8° (± 3.7°) and a conventional method mean ± SD flexion of 3.2° (± 2.5°), due to the intentional 3° flexion incorporated in the sagittal plane to prevent femoral notching in PSI planning. Computed tomography assessment for rotational alignment of the femoral components showed no difference between the two groups concerning the transepicondylar axis (*p* = 0.485) with a PSI mean ± SD external rotation of 1.5° (± 1.3°) and conventional mean ± SD external rotation of 1.5° (± 1.6°).

**Conclusion:**

PSI showed no advantage over lateralization of the femoral entry for IM guidance.

**Level of evidence:**

1

**Trial registration:**

Registered on US national library of medicine ClinicalTrials.gov (NCT02993016) on December 12^th^ 2016.

## Background

To attain optimal outcomes and longevity of total knee arthroplasty (TKA), adequate component position and alignment of the limb axis with soft-tissue balance needs to be established [1]. This can be quite challenging in the context of lateral femoral bowing. Little has been published on this subject, although higher incidence rates in the Asian populations have been reported [2]. Yau et al. (2007) reported an incidence rate of 44% for lateral femoral bowing [mean ± standard deviation (SD) 5.3° ± 3.2°] in a Chinese population [3], while Mullaji et al. reported an incidence of 18% in India (mean ± SD: 7.1° ± 2.4°) [4].

Reflecting on the outcomes of TKA, measured by pain relief, implant survivability, function improvement, and patient satisfaction, we found three interlacing factors at play [5]. Relevant patient factors included body and limb size, weight, daily activity, comorbidities, and surgical response at the psychological and physiological levels [5, 6]. Implant factors constituted component size, geometry, tribology, alignment, and position [5, 6]. Finally, surgical factors were surgeon skill and experience, duration of surgery, preparation, and implantation of the prosthesis [5, 6]. Mechanical axis restoration, soft-tissue balance, and component alignment were the three major elements required for good prosthesis implantation [5, 6].

Use of short film radiographs and conventional jigs with standard valgus cutting of the distal femur boasts a potential for errors [3]. It is a frequent practice to cut the distal femur with jigs of 5° to 6° valgus and, in fact, most instruments do not offer a wide range of jig cutting angles [7, 8]. Some studies have pointed out that the accuracy of our implant orientation will be dramatically jeopardized in cases with femoral deformities, particularly in those involving lateral bowing [3, 9]. Moreover, lateral femoral bowing leads especially to challenges in achieving soft-tissue balance due to strains on the collateral ligaments [10, 11].

In confronting this situation, we proposed the following question: is patient specific instrumentation (PSI) a superior answer for addressing such problems or can we attain adequate outcomes using conventional intermedullary (IM) instrumentation in lateral femoral bowing by planning and lateralization of the femoral entry point? Also, can the use of widely available conventional guides be extended? Our initial hypothesis leaned toward PSI in that this approach results in more superior outcomes due to its planning protocol and implementation, which is not affected by the extra-articular deformity.

## Materials and methods

This randomized controlled trial (RCT) was approved by our Institutional Review Board and based on a parallel trial design with a 1:1 allocation ratio. Two treatment methods where used and executed by the senior surgeon in all cases enrolled; of these, treatment A was primary TKA performed with conventional instrumentation with IM guides for the femur and an extramedullary guide for the tibia using a posterior-stabilized (PS) knee system (Vanguard®; Zimmer Biomet, Warsaw, IN, USA) (conventional group), while treatment B was the PSI (Signature™; Zimmer Biomet, Warsaw, IN, USA) group treated using the same Vanguard PS knee system. In each patient, one knee received treatment A and the other received treatment B by simple randomization of treatment six weeks prior to surgery. The surgeon and evaluator were blinded until the date of surgery while the patient was blinded until after the surgery of both knees. No changes throughout the trial were made. All patients enrolled agreed to join the study and signed an informed consent form. Statistical analysis was performed using Microsoft® Excel® for Microsoft 365 MSO (Microsoft Corporation, Redmond, Washington, USA) (16.0) with the analysis toolPack. All entries and statistical analysis was done by one person to preserve integrity of data.

The femoral bowing angle on the coronal plane was measured using the acute angle between the mid-endosteal canal axes drawn at the proximal and distal femoral shaft quarters. Here, the proximal femoral shaft quarter extended from 0 to 5 cm below the lesser trochanter and the distal quarter extended from 5 to 10 cm above the lateral femoral condyle’s lowest part. Femoral bowing was confirmed when the bowing was at least 5° [2, 9, 10, 12, 13].

This study was conducted from February 2017 to August 2020 in our hospital, including a total of 336 patients eligible for simultaneous or staged bilateral TKAs. All patients going for bilateral primary TKAs—whether simultaneous or staged—were screened for lateral femoral bowing using the method described by Xiaojun et al., who defined lateral femoral bowing as the crossing of the medial cortex of the femur by a line extending between the apex of the intercondylar notch and the greater trochanter of the femur [14]. All cases of lateral femoral bowing identified by screening were then measured to include bowing of at least 5° bilaterally and to exclude those who did not meet this criterion using the method described by Lasam et al. [2, 9, 15]. Lasam et al. defined femoral bowing as the angle between two lines passing through the mid-endosteal canal of the femur, where the first line is at the level of 0 and 5 cm distal to the lesser trochanter and the other is at 5 and 10 cm proximal to the lowest point of the lateral femoral condyle [2, 9, 15].

All patients booked for bilateral staged or simultaneous TKA with Kellgren–Lawrence grade 3 or 4 osteoarthritis of the knee and bilateral lateral femoral bowing of at least 5° were eligible for inclusion. Meanwhile, all patients with deformities due to trauma or tumors, previous lower-limb surgery with implants, or femoral bowing not amenable to intra-articular correction were excluded. The feasibility of intra-articular correction was judged by drawing a line perpendicular to the mechanical axes of the femur representing the distal femoral resection on long film radiographs preoperatively. If the line passed through the insertion of the collateral ligament on the distal femoral condyles, then intra-articular resection alone could not be applied to correct the pathology and, hence, the case was not a candidate for this study [7, 16, 17].

**Fig. 1:**
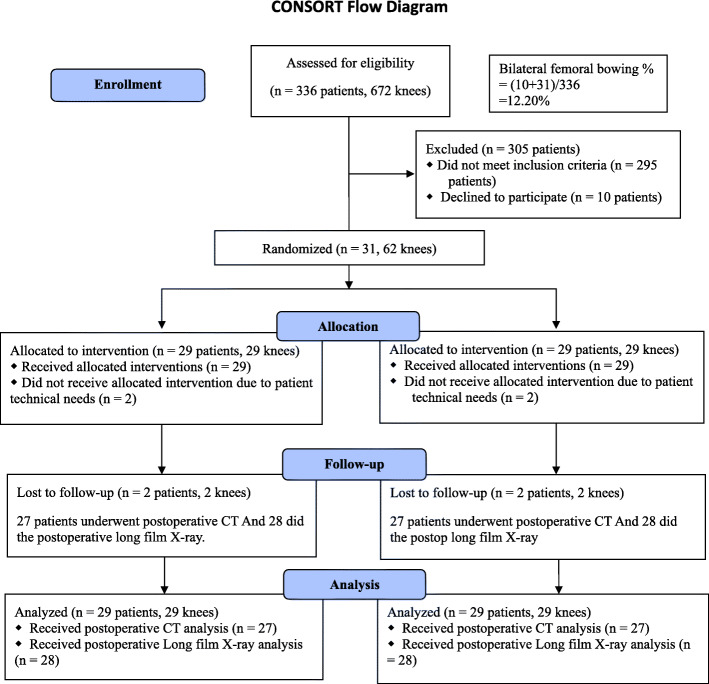
Consolidated Standards of Reporting Trials flow diagram

Among 336 patients planed for bilateral TKA assessed for eligibility, 31 patients were selected (Fig. 1). Two of these patients were subsequently excluded for severe ankylosis warranting a tibial tubercle osteotomy and PSI converted to conventional guides due to a manufacturing mistake, respectively. We obtained postoperative computed tomography (CT) scans for 27 patients and postoperative long film radiographs for 28 patients. This RCT was ended when we exceed the number of participants required as suggested by a priori power analysis.

**Fig. 2:**
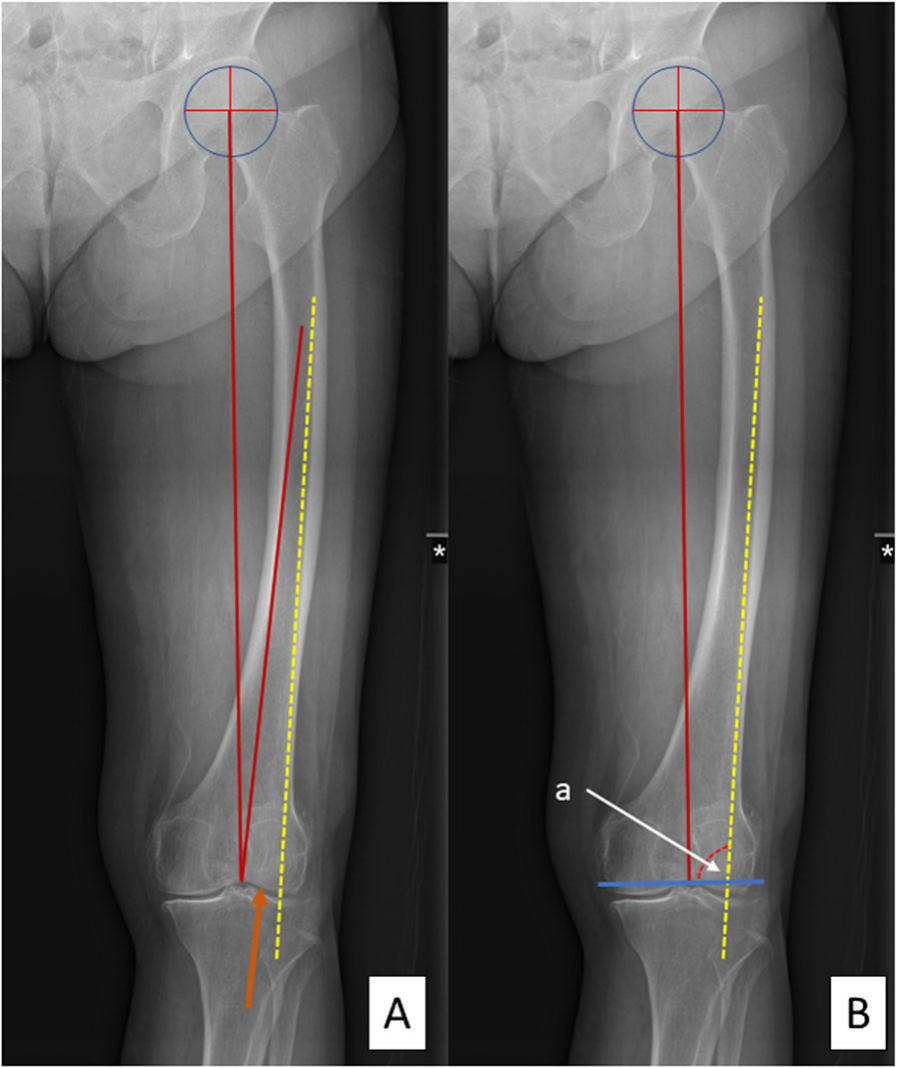
Planning for lateralization of the IM guide with lateral femoral bowing. Mechanical axis of the femur and potential IM guide axis from the apex of the intercondylar notches is marked with red lines. A yellow dotted line is drawn traversing the lateral cortex that represents the most capable path of passing through the IM canal. The lateralized entry point is in a position equivalent to half the diameter of the guide (orange arrow) (**a**). A perpendicular line to the mechanical axis is drawn (blue line), representing the distal femoral cut. The cutting angle is the product of 90° subtracted from angle “a” (**b**)

The surgical technique was standardized by the senior surgeon, who performed all operations. A neutral mechanical alignment on the coronal plane was aimed for both surgical techniques. The subvastus approach was applied in all cases. Preoperative planning using long film anteroposterior (AP) standing radiographs of the lower limb on the side receiving treatment A incorporated the following steps. First, on an AP-view long film radiograph, three lines were drawn, which were the mechanical axis of the femur, the IM guide pathway from the apex of the intercondylar notch, and the isthmus of the femur, while another line was drawn along the lateral cortex representing the most capable path passing through the IM canal. Second, the lateral entry point was marked at a distance equal to half the diameter of the IM guide medial to the lateral cortex line, doted in yellow (Fig. 2a). Third, the distance from the entry point to the apex of the intercondylar notch was noted. Finally, the cutting angle was calculated by subtracting 90 from the angle formed between the perpendicular line to the mechanical axis and the lateral cortex line (Fig. 2b).

**Fig. 3:**
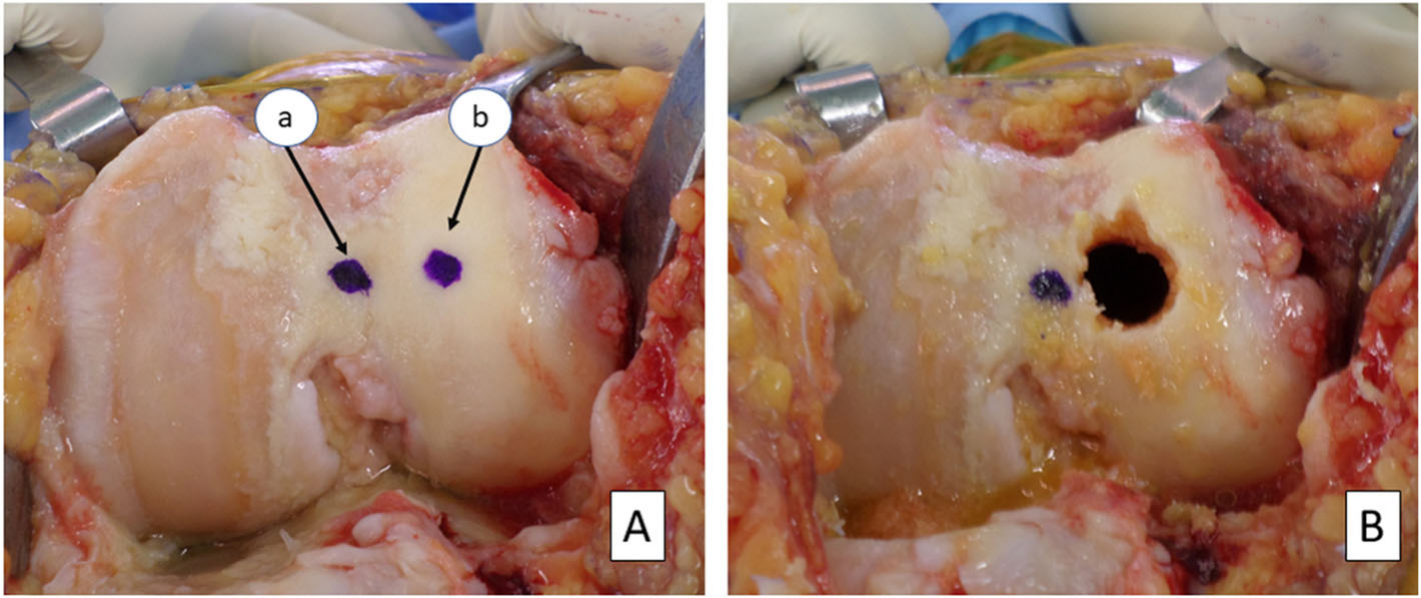
Lateralization of the entry point intraoperatively in the left knee. a marks the traditional entry point for the guide at the apex of the intercondylar notch. b represents the mark of the lateralized entry point. (**a**) the lateralized entry point is opened in a wide fashion by conically rotating the drill bit (**b**)

Intraoperative marking of the femoral guide entry point is shown in Fig. 3. First, the traditional entry point was marked; then, the distance calculated previously was used to mark the lateralized entry point as shown in Fig. 3a. Finally, the lateralized entry point was drilled in an enlarged fashion by rotating the drill bit in a conical fashion to provide wiggle space for the guide to enter (Fig. 3b). With lateralization of the femoral entry, we aimed to find the most open path for the IM guide to reach the femur’s isthmus, which, in turn, extends the utility of our instruments [12]. For the group receiving treatment B, PSI was ordered six weeks prior to surgery with a goal of neutral mechanical alignment. The process of PSI acquisition involved ordering a CT scan of the lower limb in accordance with the manufacturer’s protocol, then uploading said scan to their site and waiting for an implant proposal from their engineers, which was then approved by our senior surgeon with parameters similar to the conventional side except for the sagittal plane alignment of the femoral component, which was planned with an extra 3° flexion to avoid femoral notching. After approval by the senior surgeon, manufacturing of the PSI was completed by the company in their facilities and sent to our hospital.

**Fig. 4:**
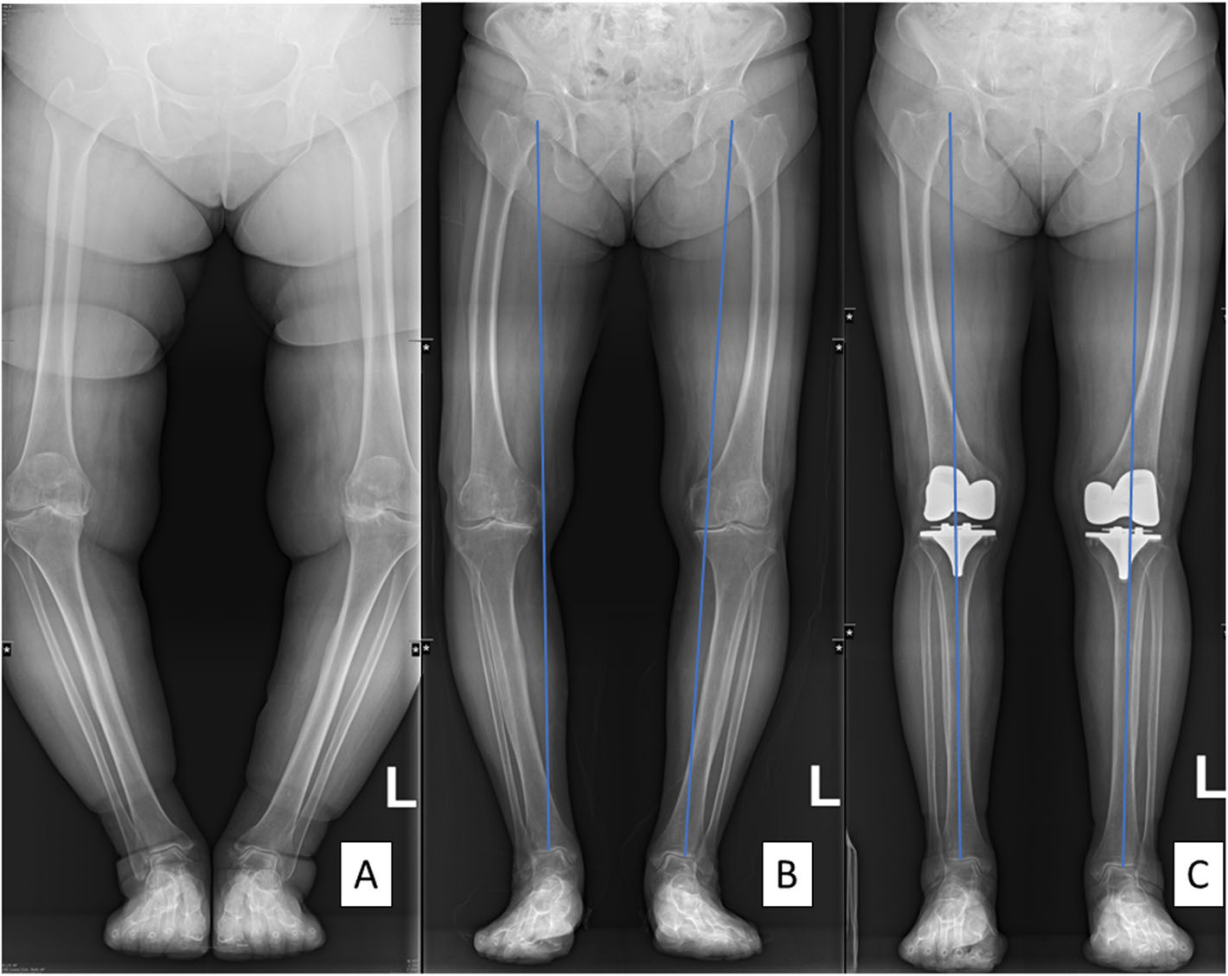
Lower limb long-film X-ray. Severe genu varum in a 57-year-old female patient with no extraarticular deformity (**a**). Bilateral severe femoral bowing seen preoperatively in an 85-year-old female patient enrolled in this study (**b**). Postoperative X-rays of patient B (**c**). The right side was treated using conventional instruments and the left side was treated using PSI

Our primary outcome measurements were defined as follows. On the long AP radiograph of the lower limbs with weight-bearing, the following points were marked to draw the mechanical axes of the lower limb: the center of the femoral head, the mid-condylar point of the distal femur, the tibial plateau center marked by the interspinous midpoint, and the tibial plafond center (Fig. 4). Each axis was defined as follows: femoral mechanical axis (femoral head-distal femur), tibial mechanical axis (tibial plateau center-tibial plafond center), and overall mechanical axis (femoral head-tibial plafond center). The hip–knee–ankle (HKA) angle was formed by the acute angle between the femoral and tibial mechanical axes, with a negative sign for varus and a positive sign for valgus [9, 18].

Meanwhile, the mechanical lateral distal femoral angle (mLDFA) was formed between the mechanical axis of the femur and the distal femoral joint line [19], the anatomic lateral distal femoral angle (aLDFA) was formed between the distal femoral mid-IM axis and the distal femoral joint line [19], and the medial proximal tibial angle (MPTA) was formed between the tibial mechanical axis and the proximal tibial joint line [19]. The joint lines for the distal femur were drawn passing through the subchondral aspect of the distal femoral condyles, while, for the proximal tibia, a line was established passing the tibial plateau through the subchondral aspect of its concavities. Additionally, the joint line convergence angle (JLCA) was formed between both knee joint lines mentioned [19] and the distal femur valgus correction angle was formed between the mechanical axis and an axis extending from the intercondylar notch until the isthmus of the femur [3]. The component alignment was measured in accordance with the Knee Society Radiographic Evaluation System and Methodology for TKA [20].

The component alignment goals for the tibial component were 90° valgus on the coronal plane and 87° on the sagittal plane, while, for the femoral component, the sagittal angle for PSI was flexed by 3°, that for the conventional side was 0°, and the coronal angle was 90° [6]. On axial CT cuts of the distal femur, the posterior condylar angle was formed between the transepicondylar axis (TEA) and the posterior condylar line. The TEA was drawn between the medial and lateral epicondylar prominences of the distal femur [21, 22]. The posterior condylar line (PCL) was defined as a line passing through the subchondral border of the most posterior aspect of the femoral condyles [21, 22].

### Statistics

A priori power analysis was performed using an online calculator provided freely by sealed envelope®. Twenty-eight patients (*n* = 56 knees) were estimated to detect for a 5% significance level with 80% confidence interval to exclude the HKA angle difference of means by 2.2 based on a SD assumption of 2.8 [23]. All data analyses involved continuous variables and regular descriptive tests were applied. A t-test assuming unequal variance was used to compare the means of the conventional to PSI side for every variable. These statistical modalities were easily available in a downloadable analytical pack by Microsoft for Excel (Microsoft Corporation, Redmond, Washington, USA).

## Results

Both groups of knees after allocation showed no significant differences concerning preoperative lateral femoral bowing, alignment, or joint orientation (Table 1). The incidence of bilateral lateral bowing in our research was 12.2% of all bilateral TKAs completed in our department. Lateral bowing for the conventional group vs. PSI group was 9.2° vs. 9.6° with *p* = 0.327. The HKA angle mean was varus − 13.1° for the conventional TKA group but varus − 12.7° for the PSI group with *p* = 0.401. Among the 29 patients, only one was male. On the right side, 13 PSI and 16 conventional TKA procedures were performed, with the opposite side receiving the inverse proportions of procedures.

**Table 1:** Preoperative lower-limb alignment and joint orientation

	Conventional (***n*** = 29)		PSI (n = 29)		
	Mean (SD)	95% CI	Mean (SD)	95% CI	***p***-value
**Lateral femoral bowing (°)**	9.2 (4.1)	7.7–10.6	9.6 (3.0)	8.5–10.7	0.327
**HKA angle (varus °)**	13.1 (5.2)	11.2–15	12.7 (6.1)	10.5–15	0.401
**mLDFA(°)**	90.9 (3.2)	89.7–92.1	90.6 (2.4)	89.7–91.4	0.328
**aLDFA(°)**	80.6 (2.3)	79.8–81.5	80.9 (2.5)	79.9–81.8	0.321
**MPTA(°)**	83.1 (3.6)	81.8 ~ 84.4	83 (4.5)	81.4 ~ 84.7	0.448
**Joint translation**	2.5 (3.2)	1.3–3.6	2.4 (3.2)	1.2–3.6	0.470
**JLCA(°)**	4.4 (3.4)	3.2–5.7	4.9 (3.1)	3.8–6.0	0.304
**PCA (°)**	6.2 (1.8)	5.6–6.9	6.2 (1.5)	5.6–6.7	0.427

*HKA* hip-knee-ankle, *mLDFA* mechanical lateral distal femoral angle, *aLDFA* anatomic lateral distal femoral angle *MPTA* medial proximal tibial angle *JLCA* joint line convergence angle *PCA* posterior condylar angle

No differences were found between postoperative alignment and component orientation for the conventional group vs. the PSI group except with respect to the sagittal orientation of the femoral component due to the preplanned 3° flexion for PSI, which was included intentionally to prevent undesired femoral notching (*p* = 0.001) (Table 2). The postoperative lower-limb alignment mean was varus 4.0° for HKA on the conventional side and varus 4.1° on the PSI side. The postoperative TEA angle for 27 patients was externally rotated 1.5° on the conventional side and externally rotated 1.5° on the PSI side (*p* = 0.485). The PSI group showed no advantage over the conventional group in terms of tourniquet time or bleeding. The conventional side showed less tourniquet time (*p* = 0.001), with a mean of 40 min.

**Table 2:** Postoperative parameters

	Conventional (n = 29)		PSI (n = 29)		
	Mean (SD)	95% CI	Mean (SD)	95% CI	***p***-value
**HKA angle (goal = 0°)**	4.0 (2.7)	3–5	4.1 (3.1)	2.9–5.2	0.459
**CFA, α (goal = 96°)**	94.5 (4.2)	93–96	95.1 (4.40)	93.5–96.7	0.298
**CTA, β (goal = 90°)**	89.4 (1.8)	88.8–90.1	90.2 (2.3)	89.3–91	0.091
**SFA, γ (goal = 0° for conventional, 3° for PSI)**	3.2 (2.5)	2.3–4.1	5.8 (3.7)	4.5–7.2	0.001
**STA, ϕ (goal = 87°)**	85.4 (3.3)	84.2–86.6	84.8 (4.5)	83.1–86.4	0.283
**PCA (goal = 0°)**	1.5 (1.6)	0.9–2.1	1.5 (1.3)	1–2	0.485
**Tourniquet time (min)**	40 (5.9)	37.9–42.2	46.5 (8.5)	43.4–49.6	0.001
**Bleeding (ml)**	150 (85.8)	118.8–181.3	162.1 (84.4)	131.4–192.8	0.296

*HKA* hip-knee-ankle, *CFA* coronal femoral angle *CTA* coronal tibial angle *SFA* sagittal femoral angle *STA* sagittal tibial angle *PCA* posterior condylar angle

No adverse events requiring additional treatment occurred in either group after surgery.

## Discussion

The most important finding was that PSI was not superior to conventional guides in treating lateral femoral bowing. Both groups showed no differences in the postoperative lower-extremity alignment and component positions.

Femoral bowing has higher incidence rates in the Asian population with some studies reporting rates as high as 44.9% [12]. In the Korean population, bowing has been reported at a rate of 11.7% [13]. Severe coronal bowing can be missed clinically, either on short film radiographs or intraoperatively, pointing out the need for long film radiographs to detect and plan the management of such cases [1, 13]. The 3° within an optimal position is the degree of accuracy needed for component cuts to achieve acceptable outcomes as reported by many investigations [7]. The use of long film radiographs in planning TKA in populations with higher rates of femoral bowing is crucial to achieve adequate orientation and avoid unexpected obstacles [7, 24]. Marked bowing, if not planned for, can compromise IM guide alignment [3]. Femoral bowing may also impact tissue balancing by influencing the femoral condylar orientation [10].

PSI is a relatively recent technology ushered in by advances in rapid prototyping technologies. A critical overview of the technology can be arranged from several angles like cost, scanning techniques, time lag, intraoperative verification, accuracy, and tissue balancing [25]. Although, some researchers claim that PSI presents a more economical option, details regarding the true overall cost of all processes involved in this approach and on whom the burden of payments lies seems to be elusive as such information is highly dependent on local regulations and settings [26, 27]. Due to governmental or insurer regulations that vary with the locality, the cost of CT scanning or magnetic resonance imaging (MRI) may be billed to the patient, which in term may disturb them due to the added cost relative to that of conventional measures [25]. Another aspect that affects the cost variability is the different treatment settings from commercially available to hospital-based systems and the need for company-specific instrumentation and program algorithms [25]. As seen from the scanning technique used in planning the procedure and modeling the jigs, some options support using CT scans and others support using MRI [25, 26]. Those who support CT imaging do so based on the simplicity of acquiring image slices in this context accurately, with no need for specialized technicians, in comparison with MRI [25, 26]. Conversely, those who support the usage of the MRI do so based on the fact that cartilage and soft tissue can be detected with this imaging modality, which is not possible with CT, making intraoperative orientation points more easily identifiable without stripping off the cartilage [25, 26]. The overall issue inherent in using scanning is that it makes an added chokepoint in the workflow, which differs from one hospital setting to another. Regarding the amount of time saved by using PSI, it is assumed that, by eliminating steps in comparison with those required for the application of IM guides, the operative time will be reduced [25, 26]. However, although reports pointing to a reduction in operative time do exist, the difference is not significant [25–27]. Further, when looking at the overall treatment process, we can observe a lag time of three to eight weeks from choosing to do PSI until acquiring the fabricated jig from the manufacturer [25]. Moreover, a learning curve does exist when using the PSI approach, reported by some to be 10 patients [25, 26]. One of the great setbacks with PSI is the absence of intraoperative verification tools, which translates to the inability to identify deviations from norms intraoperatively [25]. This has led some operators to use computer guide tools for verification purposes, which represents an added cost and time drain [25]. This is an issue especially given reports of inaccuracy of the tibial jig reaching from 60 to 70% from some manufacturers [25]. Most reports point out that PSI is as good as IM guides but not necessarily obviously better [25–28]. In the end, when considering previous points, utilizing IM guides and extending its utility will be more cost effective than the additional costs of PSI, for no orthopedic service can apply PSI without the presence of traditional guides as a backup.

Femoral bowing leads to a potential for IM guide errors due to incomplete rod insertions, culminating in a predisposition for varus deformity and malalignment. Planning helps with preemptively addressing such issues by anticipating the proper cutting jig positions [2, 9]. Significant bowing leads to a proportionate increase in the distal femoral valgus resection angle due to an increase in the mechanical axis anatomic axis angle of the femur [15]. The 5° to 6° of the distal femoral valgus angle cut is used in the majority of TKA, which most IM guide manufacturers design around [29]. The angular relationship between the femoral mechanical axis and distal femoral anatomic axis is affected by the mLDFA, femoral bowing, and knee joint varus/valgus deformity [4]. The challenge of an adequate measured resection and the gap balance required to achieve a rectangular space lies in that femoral bowing my present with a varus condyle orientation of the femur and an inclination of the tibia that necessitates more aggressive soft-tissue release [2].

IM canal distortion or blockade by hardware from previous surgery may also constitute a challenge facing conventional instrumentation [15]. Intra-articular resection and tissue balance are dependent on the deformity’s degree and distance from the knee according to Wolff et al., where, the closer to the joint and larger the deformity, the more difficulties are encountered [16, 30]. Violation of the insertion of either collateral ligament on the femoral epicondyles is a contraindication for one step intra-articular osteotomy [7]. Corrective osteotomy is applied if the distal femoral cuts will compromise the collateral ligament attachments or if deformity of greater than 20° on the coronal plane or deformity close to the joint is observed [15]. For the tibia, corrective osteotomy is applied if the deformity is greater than 30° or is close to the joint or the axis of the distal tibia passes outside the tibial plateau [15]. Around 70 to 80% of patients show an ideal component orientation with femoral bowing using IM or extramedullary guides [8]e. In theory, the PSI concept presents an advantage, but some research has proposed no advantage over other techniques exists with this method in practice [31]. Any new technology needs to meet one of the following two criteria: in comparison with existing technology, it needs to improve the efficiency or, if outcomes are similar, it needs to reduce the cost [32] as compared with conventional IM instrumentation. Some papers have reported that PSI did not reduce the surgical time [31].

The study at hand, to the best of our knowledge, is the first to investigate the usefulness of PSI in patients with lateral femoral bowing undergoing TKA. Although we took care to apply the best standards of RCT, there were limitations, including a female sex dominance, low power due to small sample size, and measurement accuracy of the lower-limb alignment. Moreover, three-dimensional imaging of femoral bowing was not conducted and clinical outcomes were not measured.

In our study, we could not discern any difference between using IM guides and PSI with regard to the implant position or lower-limb orientation in lateral femoral bowing (Fig. 4).

## Conclusion

We found that PSI had no advantage over conventional IM methods, which could be extended in use by lateralization of femoral guide entry.

## Data Availability

The datasets used and/or analyzed during the current study are available from the corresponding author on reasonable request.

## Abbreviations

AP: Anteroposterior
aLDFA: Anatomic lateral distal femoral angle
CT: Computed tomography
HKA: Hip-Knee-Ankle angle
IM: Intermedullary
JLCA: Joint line convergence angle
mLDFA: Mechanical lateral distal femoral angle
MPTA: Medial proximal tibial angle
MRI: Magnetic resonance imaging
PCL: Posterior condylar line
PSI: Patient specific instrumentation
RCT: Randomized control study
SD: Standard deviation
TEA: Transepicondylar axis
TKA: Total knee arthroplasty
